# Epidemiological characteristics of six common respiratory pathogen infections in children

**DOI:** 10.1128/spectrum.00079-25

**Published:** 2025-05-22

**Authors:** Zheng Tang, Huihui Fan, Yaling Tian, Qingsong Lv

**Affiliations:** 1 Department of Transfusion Medicine, The Central Hospital of Yongzhou, Yongzhou, China; 2 Department of Transfusion Medicine, Yongzhou Hospital Affiliated to University of South China, Yongzhou, China; 3 Department of Clinical Laboratory, The Central Hospital of Yongzhou, Yongzhou, China; ICON plc, London, United Kingdom

**Keywords:** acute respiratory infection, children, human rhinovirus, *Mycoplasma pneumoniae*, adenovirus, influenza, respiratory syncytial virus

## Abstract

Respiratory infections are among the leading causes of hospitalization and death among children worldwide. Traditional diagnostic methods lack the sensitivity and timeliness needed for early clinical diagnosis and treatment. This retrospective study analyzed respiratory pathogen results of 15,397 pediatric patients at Yongzhou Central Hospital between June 2023 and May 2024, with emphasis on age-specific and seasonal variations. Pathogens detected included influenza A (FluA), influenza B (FluB), respiratory syncytial virus (RSV), adenovirus (ADV), human rhinovirus (HRV), and *Mycoplasma pneumoniae* (MP). The overall positivity rate was 77.0%. Single respiratory pathogen infections were detected in 52.0% of cases, while 25.0% had mixed infections. HRV exhibited the highest positivity rate (32.4%), followed by MP (20.9%), ADV (19.2%), RSV (14.1%), FluA (11.5%), and FluB (8.3%). Infection rates for FluA, FluB, and MP increased with age, peaking at 82.8% in school-aged children. RSV detection was the highest in infants (27.2%). ADV was most common in the preschool group (26.2%). Infection rate was the highest in winter (82.1%), followed by that in spring (76.7%). FLuA and FluB had a peak in winter. MP infections surged in summer (32.4%), and HRV is common in four seasons, with its prevalence peaking in spring and autumn. The incidence of respiratory pathogen infections among children varied according to age distribution and seasonal fluctuations. The findings provide a basis for accurate diagnosis, treatment, prevention, and control of these infections.

**IMPORTANCE:**

By analyzing the data of 15,397 cases of respiratory tract pathogens in children, the epidemic characteristics of six common respiratory tract pathogens in children of different ages and seasons were more comprehensively understood in this region. Proactive prevention and control measures should be taken in advance for their dominant pathogens in different months, such as community- and school-based health education, based on the epidemic and pathogenic characteristics of different pathogens, along with timely diagnosis and treatment to reduce the risk of transmission.

## INTRODUCTION

Acute respiratory infections (ARIs) are a significant burden on healthcare systems worldwide, accounting for high morbidity and mortality rates in children (1, 2). ARIs are broadly categorized into upper respiratory tract infections (URTIs) and lower respiratory tract infections (LRTIs). The pathogens responsible for respiratory tract infection in children are diverse, including viruses, typical bacteria (e.g., *Streptococcus pneumoniae*), atypical bacteria (including *Mycoplasma* and *Chlamydia* species), and fungi, and their distribution varies with age, season, region, and detection methods. Recent studies have identified viruses as the predominant cause of respiratory infections in children, accounting for approximately 60–70% of cases, with influenza, RSV, HRV, human metapneumovirus (HMPV), ADV, and parainfluenza viruses (PIV) being the most prevalent (3, 4). Bacterial pathogens, including *Streptococcus pneumoniae*, *Haemophilus influenzae*, MP, *group A Streptococcus*, and *Staphylococcus aureus*, cause the remaining significant proportion of cases (3, 5, 6).

The symptoms of respiratory infections caused by different respiratory pathogens are often similar, and traditional diagnostic methods, such as culture, microscopic examination, and immunoassay, lack the sensitivity and timeliness needed for early clinical diagnosis and treatment. Therefore, using multiple fluorescent polymerase chain reaction (PCR) techniques to rapidly identify respiratory pathogens is crucial for timely clinical diagnosis, disease prevention and control, and rational use of antibiotics. This study retrospectively analyzed the nucleic acid test results of six common respiratory pathogens in 15,397 children in the Yongzhou urban area to assess the epidemiology of respiratory infections, including age and season variations, and provide a basis for clinical diagnosis and appropriate antibiotic use.

## MATERIALS AND METHODS

### Clinical data of the patients

From June 2023 to May 2024, a retrospective analysis was conducted on the nucleic acid test results for six respiratory pathogens in 15,397 children (including outpatient and inpatient, aged <14 years) with suspected ARIs who visited pediatricians at the Central Hospital of Yongzhou. Among them, there were 8,950 males and 6,447 females, with an average age of 4.6 years. All repeated tests from the same patient within 30 days were removed, and all collected data excluded the patient’s name or other private information.

### Collection, preservation, and transportation of specimens 

Throat or nasopharyngeal swab was collected by a trained physician using a disposable sterile swab (Maidi, Shenzhen, China), placed in a matching sterile viral transport medium (VTM) tubes (X1004, Shengxiang, Hunan, China), and immediately sent to the clinical laboratory for detection. In cases where immediate delivery was unfeasible, the sample was stored at 4°C for 48 h.

### Pathogen nucleic acid detection

Respiratory pathogen nucleic acids were extracted using the manufacturer’s matched extraction reagents on an automated magnetic bead-based instrument (Model Natch 32A; Reagent Lot S20025, Shengxiang, Hunan, China), followed by detection with the manufacturer’s six-respiratory-pathogen nucleic acid diagnostic kit (S3066E-24), which targets common respiratory pathogens (influenza A/B, RSV, ADV, HRV, and MP). The target genes of these pathogens were identified as the matrix protein (M gene), large polymerase (L gene), hexon gene, 5′-untranslated region (5′-UTR), and cytadhesin protein (P1 gene). The endogenous reference gene was human GAPDH. Multiple real-time fluorescence quantitative PCR detection technologies were applied using the SLAN-96P fully automatic medical PCR analysis system (Hongshi, Shanghai, China) for the rapid detection of respiratory pathogen nucleic acids based on changes in fluorescence signals. All other procedures were performed according to the manufacturers’ instructions.

### Statistical analysis

Enumeration data were calculated using frequency analysis. IBM SPSS Statistics software (version 21.0) and χ test were used to compare multiple rates between groups, and *P* < 0.05 was considered statistically significant.

## RESULTS

### Overall pathogen detection

Among 15,397 cases analyzed, 77.0% (11,860) tested positive for at least one pathogen. This included cases of infection with a single pathogen 52.0% (8,010) and multiple mixed infections 25.0% (3,850). Among the mixed infections, human rhinovirus (HRV) combined with *Mycoplasma pneumoniae* (MP) was the most common, accounting for 17.4% (669). The second most common pattern was HRV coinfection with ADV 16.8% (647) or RSV 9.2% (356). The overall positive detection rate for each pathogen was statistically significantly different (*P* < 0.001, Table 1), with HRV having the highest positivity rate of 32.4% (4,984), followed by MP, adenovirus (ADV), respiratory syncytial virus (RSV), influenza A (FluA), and influenza B (FluB).

**TABLE 1:** Positive detection of six respiratory pathogens in children [*n* (%)]^*a*^

Pathogens	Total positive	Single positive	Mixed positive	*Χ* ^2^	*P*
FluA	1,768 (11.5)	901 (5.9)	867 (5.6)	*χ*^2^ = 3,185.1	<0.001
FluB	1,282 (8.3)	740 (4.8)	542 (3.5)		
RSV	2,177 (14.1)	1,198 (7.8)	979 (6.3)		
HRV	4,984 (32.4)	2,465 (16.0)	2,519 (16.4)		
ADV	2,952 (19.2)	1,210 (7.9)	1,742 (11.3)		
MP	3,218 (20.9)	1,495 (9.7)	1,723 (11.2)		

^*a*^ FluA, influenza A; FluB, influenza B; RSV, respiratory syncytial virus; ADV, adenovirus; HRV, human rhinovirus; MP, *Mycoplasma pneumoniae.*

### Comparison of respiratory pathogen infection in children of different sexes

**Fig 1:**
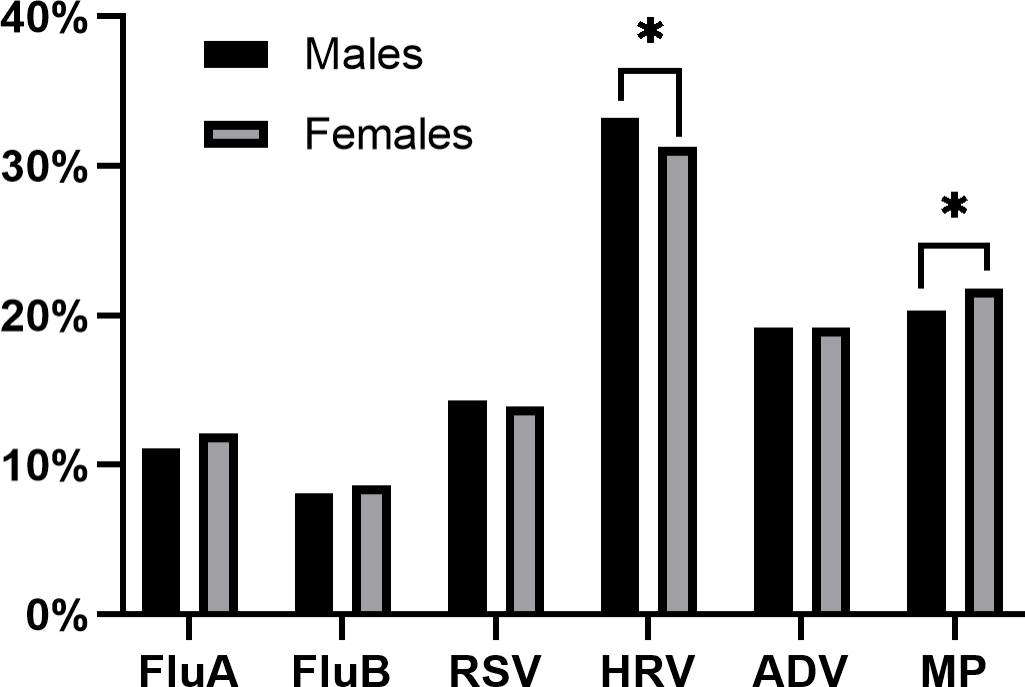
Positivity rates of six pathogens in different sexes.

Among the 11,860 positive cases, 58.3% (6,911) were males and 41.7% (4,949) were females. There was no significant difference in the overall detection rate of pathogens between different sex groups (χ2 = 2.3, *P* > 0.05). However, when comparing sex differences in pathogen positivity, only HRV and MP showed statistically significant differences (*P* < 0.05; Fig. 1).

### Comparison of respiratory pathogen infection in children of different ages

**Fig 2:**
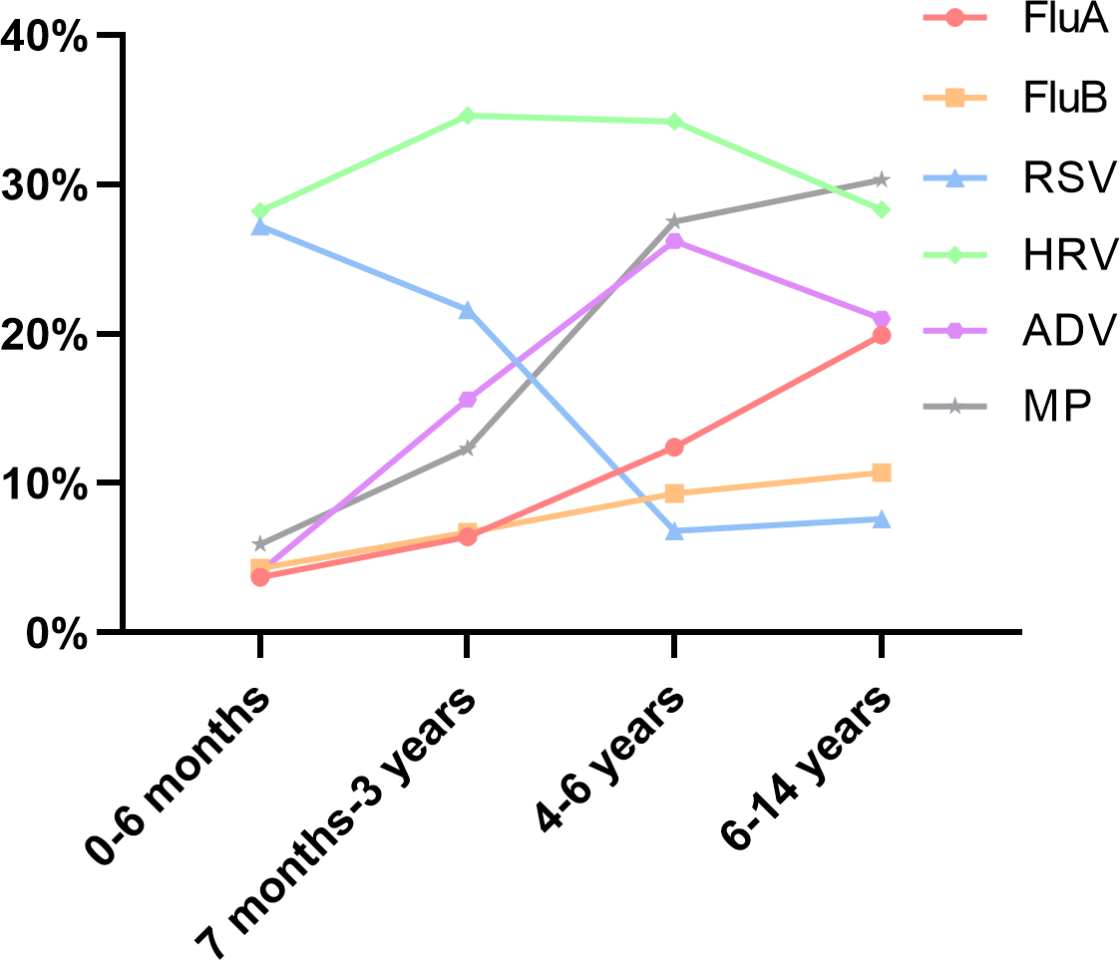
Positivity rates of six pathogens in different age groups.

All patients were classified into four age groups: infants (0–6 months), toddlers (7 months–3 years old), preschoolers (4–6 years old), and school-aged children (7–14 years old). The cohort comprised 1,094 infants, 5,885 toddlers, 4,304 preschoolers, and 4,114 school-aged children. Pathogen positivity rates exhibited significant variation across age groups (*P* < 0.001; Table 2). As shown in Fig. 2, the infection rates for FluA, FluB, and MP increased with age. In contrast, ADV peaked in the preschool group. RSV showed the highest positivity rates among infants (27.2%) and toddlers (21.6%). Notably, HRV demonstrated consistently high infection rates across all age groups.

**TABLE 2:** The positivity rate of six pathogens in different age groups [*n* (%)]

Pathogens	0–6 months *n* = 1,094	7 months–3 years *n* = 5,885	4–6 years *n* = 4,304	7–14 years *n* = 4,114	*Χ* ^2^	*P*
FluA	40 (3.7)	378 (6.4)	532 (12.4)	818 (19.9)	503.0	<0.001
FluB	47 (4.3)	394 (6.7)	402 (9.3)	439 (10.7)	79.2	<0.001
RSV	298 (27.2)	1,272 (21.6)	294 (6.8)	313 (7.6)	759.4	<0.001
HRV	309 (28.2)	2,038 (34.6)	1,472 (34.2)	1,165 (28.3)	59.7	<0.001
ADV	45 (4.1)	917 (15.6)	1,126 (26.2)	864 (21.0)	353.6	<0.001
MP	65 (5.9)	722 (12.3)	1,183 (27.5)	1,248 (30.3)	747.8	<0.001

### Seasonal epidemiological characteristics of respiratory tract infection pathogens 

**Fig 3:**
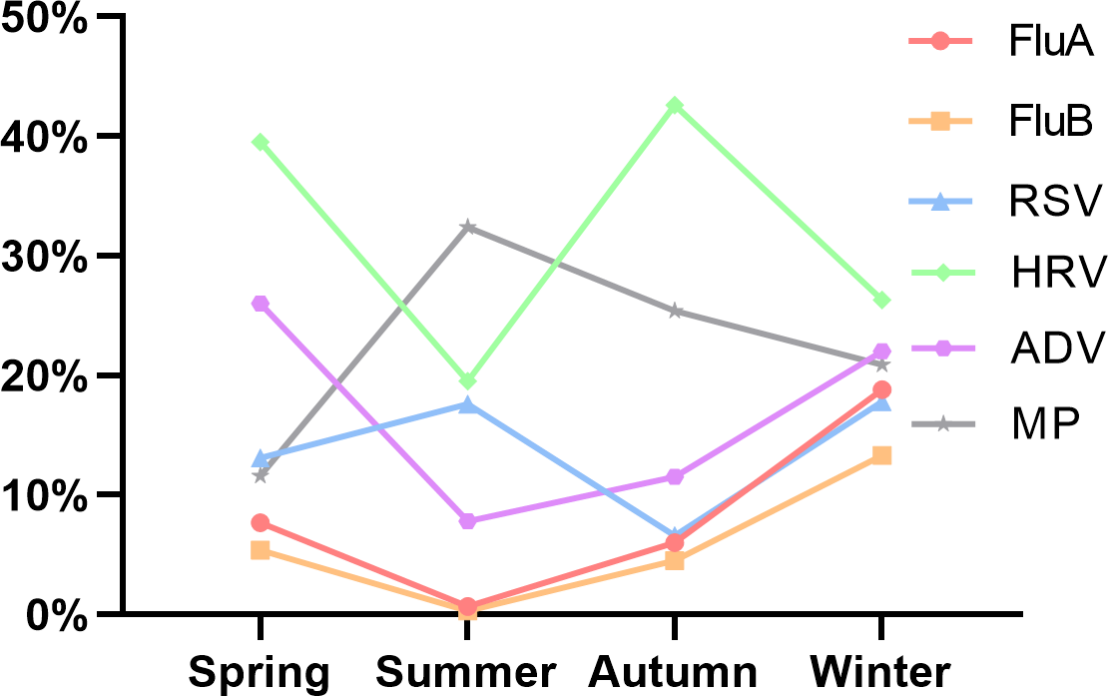
Positivity rates of six pathogens in different seasons.

The traditional seasonal division in the northern hemisphere comprises spring (March to May), summer (June to August), autumn (September to November), and winter (December to February). The overall pathogen detection rate varied significantly by season (*P* < 0.001), peaking in winter 82.1% (5,793) and spring 76.7% (2,617) (Table 3). Distinct seasonal patterns were observed for individual pathogens (Table 4; Fig. 3): HRV exhibited bimodal peaks, with high prevalence in both spring (39.5%, 1,347) and autumn (42.6%, 1,515); MP infections surged in summer (32.4%, 443), representing its annual apex; FluA/FluB were predominantly detected in winter; ADV circulated at elevated levels during winter and spring; and RSV showed dual peaks in summer (17.6%, 240) and winter (17.8%, 1,256).

**TABLE 3:** Total respiratory pathogen infection in different seasons [*n* (%)]

Seasons	Single positive	Mixed positive	Negative	Total	*Χ* ^2^	*P*
Spring	1,825 (53.5)	792 (23.2)	797 (23.3)	3,414	390.7	<0.001
Summer	719 (53.6)	166 (12.1)	482 (35.3)	1,367		
Autumn	1,789 (50.3)	776 (21.8)	995 (27.9)	3,560		
Winter	3,677 (52.1)	2,116 (30.0)	1,263 (17.9)	7,056		
Total	8,010 (52.0)	3,850 (25.0)	3,537 (23.0)	15,397		

**TABLE 4:** Positivity rates of six pathogens in different seasons [*n* (%)]

Pathogens	Spring *n* = 3414	Summer *n* = 1367	Autumn *n* = 3560	Winter *n* = 7056	*χ* ^2^	*P*
FluA	262 (7.7%)	9 (0.7%)	213 (6.0%)	1,284 (18.2%)	625.2	<0.001
FluB	183 (5.4%)	4 (0.3%)	160 (4.5%)	935 (13.3%)	447.6	<0.001
RSV	446 (13.1%)	240 (17.6%)	235 (6.6%)	1,256 (17.8%)	260.9	<0.001
HRV	1,347 (39.5%)	267 (19.5%)	1,515 (42.6%)	1,855 (26.3%)	469.1	<0.001
ADV	886 (26.0%)	107 (7.8%)	408 (11.5%)	1,551 (22.0%)	387.3	<0.001
MP	397 (11.6%)	443 (32.4%)	903 (25.4%)	1,475 (20.9%)	329.9	<0.001

### Positivity rate of six pathogens in each month

**Fig 4:**
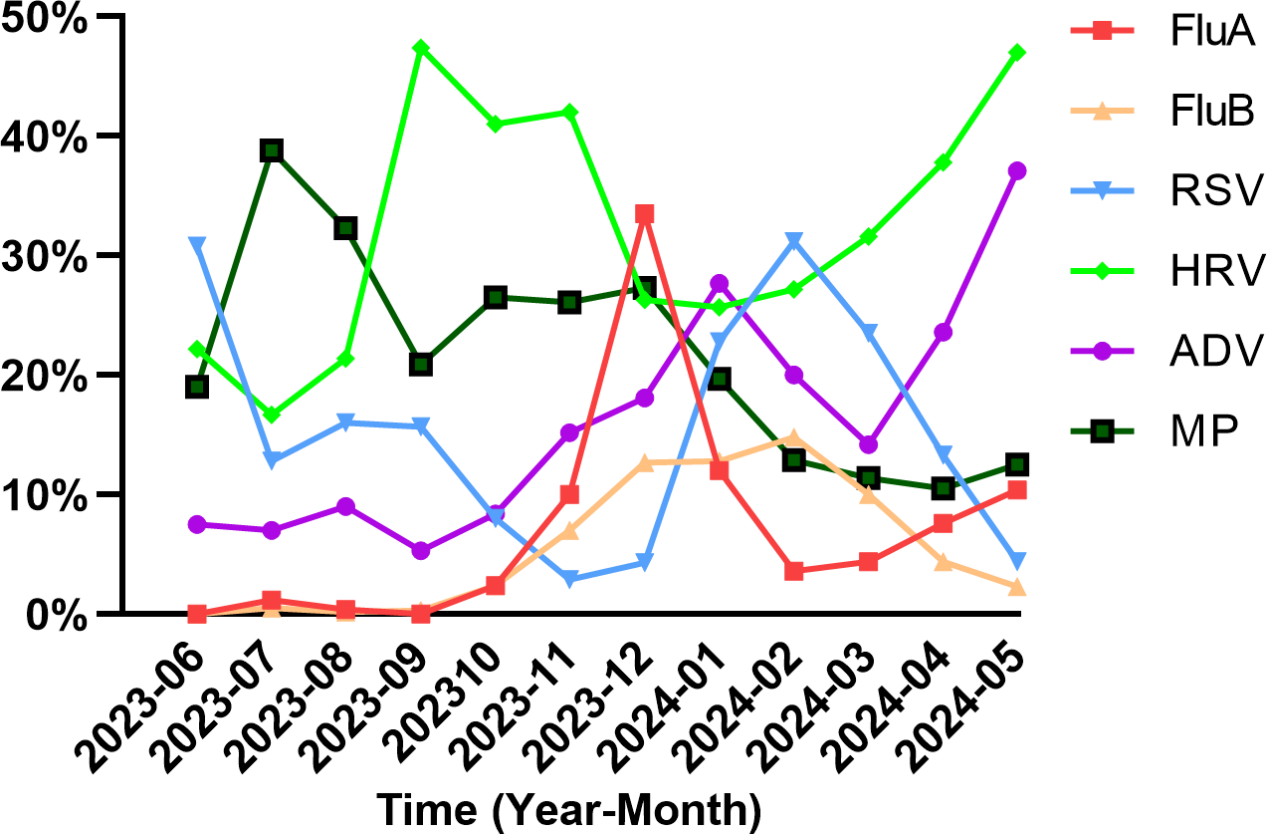
Positivity rates of six pathogens in each month.

To assess the monthly epidemic characteristics of these six pathogens, data from June 2023 to May 2024 were grouped, and the monthly positivity rates for each pathogen were analyzed. As shown in Fig. 4, the positivity rate of HRV is consistently high across all months, with two peaks in May and September. The highest positivity rates for MP occurred in July, ADV in January, FluA in December, and FluB and RSV in February.

## DISCUSSION

Respiratory infections are among the leading causes of hospitalization and death among children worldwide (2, 7). Common pathogens comprised FluA, FluB, RSV, HRV, ADV, and MP, among others. This study analyzes large-scale clinical data to examine epidemiological trends by age, sex, and season, providing critical evidence for targeted prevention strategies in children.

Notably, among 15,397 children with acute respiratory infection, 11,860 (77%) were positive for respiratory pathogen nucleic acids, consistent with previous findings in other Asian countries (8, 9). Positivity rates for HRV, MP, ADV, RSV, FluA, and FluB were 32.4%, 20.9%, 19.2%, 14.1%, 11.5%, and 8.3%, respectively. Although these detection rates were generally consistent with epidemiological data from other Chinese regions (10–12), notable differences emerged when compared with global patterns. Not only did European cohorts show higher RSV prevalence (13), but children aged <5 years in tropical areas demonstrated significantly higher ADV infection rates (14, 15).

Our study showed significant variation in infection rates regarding age. With increasing age, FluA, FluB, and MP showed an increasing trend. Chen B. reported that the infection rate of MP in children increased significantly with age, particularly in school-aged children (16), which is consistent with our findings. Crowded settings, such as kindergartens and schools, are likely to facilitate the spread of pathogens. Particularly in winter, when children spend more time indoors, poor air circulation increases the risk of pathogen transmission (17, 18). ADV showed the highest infection rate in the preschool group, likely due to three synergistic factors: (i) environmental persistence of its non-enveloped capsid (surviving >1 month on surfaces vs days for enveloped viruses) (19), (ii) high hand-to-mouth contact rates in this age group (a median of 10 episodes/hour) (20), and (iii) limited efficacy of routine hand hygiene against non-enveloped viruses (21). These findings underscore the need for enhanced surface disinfection protocols in preschool settings. Moreover, this study confirmed that the positivity rate of MP in female children was significantly higher than that in male children (16), although the reasons require further research.

RSV, a negative-sense, single-stranded RNA virus, causes seasonal respiratory infections that typically peak in winter in temperate climates and during rainy seasons in tropical areas (22). Despite decades of vaccine research, RSV continues to cause substantial morbidity and mortality in infants, immunocompromised individuals, and older adults (23). Our study revealed that RSV exhibited the highest detection rates in infants (27.2%), followed by toddlers (21.6%), significantly exceeding rates in a prior report (<1 year: 10.6%; 1–3 years: 10.2%) (24). RSV infections impose a high burden of disease in healthy young children in the community (25, 26). This is primarily attributed to immunological immaturity, characterized by the decline of maternal antibodies after 6 months of age, insufficient endogenous IgA/IgM production in young children (27, 28), undeveloped respiratory tracts, and the relatively narrow, delicate mucosa, which make them highly vulnerable to the virus (29). In contrast to HRV, which is frequently detected in both symptomatic and asymptomatic individuals, a positive RSV test result is often clinically significant, independent of viral quantity (30). Despite recent breakthroughs in adult RSV vaccines (Arexvy [31] and Abrysvo [32]), the development of pediatric RSV vaccines remains an urgent unmet need, particularly for infants under 6 months, who bear the highest burden of severe disease.

The difference in the positivity rates for each season was statistically significant. The highest infection rate was 82.1% in the winter, followed by 76.7% in the spring. In winter, the detection rate of influenza virus (FluA and FluB) increases, as reported in previous studies (33, 34). The A(H3N2) and B/Victoria lineage viruses became the prevailing strains during the 2023 influenza season in December (35), with China recording two distinct winter and spring influenza epidemics for the first time. The patterns, timing, and intensity of influenza epidemics changed after the COVID-19 pandemic, diverging significantly from historical seasonal trends (35).

Consistent with established epidemiological patterns (36), our data confirmed the year-round circulation of HRV with bimodal peaks in spring (39.5%) and autumn (42.6%), while also revealing distinct sex-specific prevalence differences (Fig. 1). This bi-seasonal pattern may be related to the prevalence of different subtypes. HRV-C type peaks in autumn in most temperate or subtropical countries, whereas HRV-A type peaks in spring (37). Moreover, the infection rate of MP was high in summer, autumn, and winter, which is consistent with the overall epidemic trend of MP in 2023 (38). Data from Chongqing showed an upward trend in the positive percentage of MP during April 2023, reaching a high positivity rate of 67.8% (12). In May 2023, the positivity rate of MP in Beijing began to increase significantly and increased sharply from October to November (39). Similarly, our data revealed an increase in MP positivity in June, reaching a peak of 38.8% in July and then stabilizing at an average rate of 26.5% until December. Professors Yu and Zhou’s post-pandemic surveillance revealed ST3 and ST14 as China’s dominant MP genotypes in 2023, with 89.2% of these strains carrying the A2063G mutation in 23S rRNA—a key determinant of macrolide resistance (40). This phenomenon can be attributed to the impact of COVID-19 infection on the immune system of the population, the changes in the microbiome, and the interaction and infection between SARS-CoV-2, influenza, and MP. The results also closely align with the immune debt theory (41).

Our study confirmed that ADV exhibited distinct seasonal peaks in winter and spring, aligning with previous epidemiological reports (42). Although most primary ADV infections in immunocompetent children are self-limiting, their clinical presentation—characterized by high fever, pharyngitis, and conjunctivitis—often mimics bacterial infections and contributes to misdiagnosis of bacterial coinfections and subsequent inappropriate antibiotic use (43). A 2023 meta-analysis estimated that 30.9–44.3% of pediatric respiratory tract infection cases received unnecessary antibiotics (44). These findings highlight the imperative for implementing rapid molecular diagnostics (e.g., multiplex PCR) in clinical practice. Timely pathogen detection could significantly reduce antibiotic misuse, thereby mitigating antimicrobial resistance (AMR) risks.

Our study has some limitations. First, we failed to identify a detectable etiology in >23% of the cases. We only tested six of the numerous respiratory pathogens. Second, there are deficiencies in the nucleic acid detection methodology. The nucleic acid amplification method has high sensitivity and a long-time window for the detection of nucleic acids. A positive result cannot clearly indicate whether the pathogen is responsible for the current infection or if it represents residual viral nucleic acid from a previous infection, nor can it confirm whether the respiratory pathogen is still infectious (30). Finally, our study cohort may not be fully representative of the general population due to potential selection bias or unmeasured confounding factors. Thus, the external validity of our findings requires further validation in more diverse populations.

Acute respiratory infections pose a significant societal burden, highlighting the need for timely influenza vaccinations and active development of effective respiratory syncytial virus and MP vaccines. Moreover, multiple fluorescent PCR technology is necessary for the detection of pathogens in children’s respiratory tract, which can detect pathogens as early as possible to guide clinical medication and avoid the abuse of antibiotic use. In the future, in addition to expanding the research scope and extending the research time, we will further combine clinical diagnosis and treatment information and compare other detection technologies, such as mNGS, for comprehensive analysis. Meanwhile, we will recruit asymptomatic healthy volunteers to collect throat swabs for detecting the carriage rates of common respiratory pathogens (e.g., adenovirus and rhinovirus).
